# Human Herpesvirus 8 and Host-Cell Interaction: Long-Lasting Physiological Modifications, Inflammation and Related Chronic Diseases

**DOI:** 10.3390/microorganisms8030388

**Published:** 2020-03-11

**Authors:** Fabrizio Angius, Angela Ingianni, Raffaello Pompei

**Affiliations:** Department of Biomedical Sciences, Section of Medical Microbiology, University of Cagliari, 09124 Cagliari, Italy; ingianni@unica.it (A.I.); rpompei@unica.it (R.P.)

**Keywords:** virus–host interaction, cell metabolism, virus infection, human herpesvirus 8, diabetes type 2

## Abstract

Oncogenic and latent-persistent viruses belonging to both DNA and RNA groups are known to cause serious metabolism alterations. Among these, the Human Herpesvirus 8 (HHV8) infection induces stable modifications in biochemistry and cellular metabolism, which in turn affect its own pathological properties. HHV8 enhances the expression of insulin receptors, supports the accumulation of neutral lipids in cytoplasmic lipid droplets and induces alterations in both triglycerides and cholesterol metabolism in endothelial cells. In addition, HHV8 is also known to modify immune response and cytokine production with implications for cell oxidative status (i.e., reactive oxygen species activation). This review underlines the recent findings regarding the role of latent and persistent HHV8 viral infection in host physiology and pathogenesis.

## 1. Introduction

The Human Herpesvirus 8 (HHV8), also known as Kaposi’s sarcoma-associated Herpesvirus (KSHV), is the most recently identified Herpesvirus found in tissue samples from immunodeficiency syndrome-associated Kaposi’s Sarcoma (KS) lesions by Chang et al. [1]. Following its discovery, HHV8 was also identified in other lymphoblastic diseases, namely primary effusion lymphoma (PEL) and Multicentric Castleman disease (MCD) [2]. Although KS is a rare disease, it has become more frequent in acquired immune deficiency syndrome (AIDS) and immunosuppressed patients, such as transplantation recipients [3]. Together with the Epstein Barr virus (EBV), with which it shares several features, HHV8 has a specific tropism for B-lymphocytes and endothelial cells [3]. Just like other Herpesviruses, HHV8 is characterized by a biphasic life cycle consisting of an acute phase during primary infection and a subsequent latent phase that involves the expression of very few genes and which may persist throughout the life of the host [4]. Although a limited number of antigens are expressed during latency, HHV8 efficiently accomplishes some important biological functions, including modifying cellular physiology, with the relevant biochemical and pathological implications that occasionally lead to typical malignancies such as KS and PEL. Because of this, the mechanisms that control virus latency may represent a crucial target for the eradication of the latent infection and for the design of specific drugs that could cure the typical chronic HHV8-related disorders. Although the virology, molecular biology and pathogenicity of HHV8 have been properly dealt with by others [2,3,5,6,7], this review wishes to mainly focus on lesser known aspects of HHV8 infection by remarking on the recent findings related to host metabolic alterations associated with the latent infection that, in turn, confers the specific behaviour and viral properties at the base of the pathogenicity.

## 2. Virology and Molecular Biology of HHV8

HHV8 is a DNA virus with a 170 Kb double-stranded genome that comprises 75 genes; some of these are also common to other Herpesviruses while others, identified from K1 to K15, are HHV8-specific. In addition, apart from several viral microRNAs found to be expressed during the latent phase [8,9,10,11,12,13], a fair number of HHV8 genes code for proteins that are homologous to cell proteins, a phenomenon known as “gene pirating” [14]. HHV8, which belongs to the *Rhadinoviridae* genus in the *Gammaherpesvirinae* subfamily, has B-lymphocytes as the cell reservoir. Thus, several cell lines of lymphatic origin (e.g., BC3 and BCBL1) obtained from patients suffering from pleural lymphoma have been extensively used as a virus source for research on exposition to phorbol ester (TPA), which induces the lytic phase and the subsequent production of infectious viral particles. HHV8 does not show a clear cytolytic effect during the lytic phase and thus, molecular methods are needed for its detection (e.g., PCR amplification of specific genes such as the latency factor LANA, or immunostaining of the surface antigen K8.1) as detailed by Gao et al. [15], who also set up a very efficient cellular model of infection and viral lytic replication using the BAC36 strain and human umbilical vein endothelial cells (HUVEC). This model allowed the lytic-latent phases to be defined, showing that virus production starts at day 1–2 and lasts until 10–15 days post infection, after which it enters into a latent state as an episome bound to the cellular DNA-associated histones where it could remain for the entire lifespan of the host [3,15,16]. During the latent infection, HHV8 induces significant alterations in cell physiology and biochemistry including the modification of cellular permeability, resistance to toxic drugs, increased resistance to stress conditions, enhancement of glycolysis and increased expression of insulin receptors (IRs) [17,18,19]. All these “acquired” properties and conditions may lead to cell transformation and oncogenesis such as KS and neoplastic induction of lymphoma cells [16,20]. Unfortunately, during the latent phase, HHV8 is not blocked by conventional anti-Herpes drugs, such as the nucleoside analogous acyclovir, since they specifically inhibit the virus production occurring in the lytic phase. However, several compounds have recently been described as being able to affect the latency of Herpesviruses [21]. In particular, Paul et al. [22] demonstrated that a known sulfone drug, namely nimesulide, can affect HHV8 latency by interacting with its binding to cell DNA. In addition, some sulfonamide drugs have recently been shown to be able to interfere with the binding of LANA to the cellular DNA [23]. It is worth noting that several other DNA (e.g., Adenoviruses and Herpesviruses) and RNA (e.g., Rubella, Dengue virus, Enteroviruses, Hepatitis C virus and HIV) viruses were found to be able to affect the host’s metabolism favouring not only the onset of tumours but also some other chronic diseases, namely metabolic syndrome and diabetes [4,24,25,26,27,28,29].

### 2.1. Lytic HHV8 Infection

The lytic phase of HHV8-infection, which permits the virus to actively replicate with the efficient production of progeny virions, requires the consecutive expression of the immediate-early proteins mainly including transcription factors and regulators, followed by the early and the late proteins, which allow viral genome replication and complete virion morphogenesis. Among these, the viral interferon regulatory factor-1 (vIRF-1), encoded by *orfK9* and mainly produced during the lytic phase of viral replication, exhibits an opposing activity to the host’s interferon inhibiting virus-induced apoptosis [30,31,32,33,34,35]. Moreover, an important “pirated” factor is the viral GPCR (similar to cell G-protein-coupled receptor) transcribed by *orf74* in the lytic phase of HHV8. vGPCR is fundamental for endothelial cell transformation and angiogenesis in KS [14,36,37,38,39,40]. Furthermore, it has been suggested that two out of the 25 microRNAs of HHV8, namely miR-K12-10 and KSHV-miR-K12-12, which are expressed more during the lytic phase than in the latent one, play an important role during viral replication or during the initial step in de novo infections [41].

### 2.2. Latent HHV8 Infection

In the latent state the HHV8 genome is bound to cell DNA in an episome form (defined as cell-virus tethering) which involves several factors, namely cellular histones, viral latency-associated nuclear antigen (LANA) and others, such as cell protein 53 (p53) and heat shock proteins [2,20,42]. Although the latent virus expresses a limited number of genes, these are extremely important in maintaining the latent state and also in inducing oncogenic transformation. Among these, a crucial role is played by the LANA, encoded by *orf73*, which is fundamental for maintaining the latent state during virus infection in permissive cells, by means of its interaction with the p53 regulatory cell protein, hence affecting cell reproduction and apoptosis. LANA has been reported as being one of the most important proteins for cell transformation and oncogenesis in endothelial and epithelial cells [43]. Besides LANA, the *orf71* encodes for viral FLICE inhibitory protein (vFLIP) that shows homology with the cellular FLIP (Fas-associated beta-convertase enzyme). vFLIP inhibits apoptosis and has an important role in cell transformation and tumour induction. vFLIP also acts as a chaperone for binding the heat shock protein 90 (HSP90) to the LANA-cell DNA complex [44,45,46]. Kaposines A, B and C are viral proteins produced by *orfK12* mainly during the viral latent phase, with Kaposin B being the most abundant as compared to A and C. Kaposines are believed to play a role in cell transformation and cytokine functions [47]. The *orf72* encodes for viral cyclin (vCyclin) and *orf1* for K1, which is a glycoprotein involved in cell membrane function [48,49]. K1 is a very important protein for modifying host cell metabolism, since it enhances the production of angiogenic factors such as VEGF (vascular-endothelial growth factor) and activates the PI3K/AKT/mTOR cascade, thus playing a significant role in oncogenesis and in the production of the typical angiogenic lesions of KS [50,51]. Viral Interleukin-6 (vIL-6) encoded by *orfK2*, which is a homolog of cellular IL-6, is important for viral oncogenesis and the expression of angiogenic factors [52]. Moreover, during latency, the genes for the K1, K15 (*orf75)* proteins and vIL-6 are expressed in low amounts as some microRNAs [9,10]. HHV8′s miRNAs, transcribed from a ~4 kb noncoding sequence located between the *orfK12* and *orf71* genes, have been reported to inhibit the apoptosis of latently-infected cells by targeting apoptotic genes, and to enhance immune evasion and viral pathogenesis by regulating host immune responses [41]. Finally, HHV8 can occasionally re-enter the lytic phase from the latent state by means of the activation of the replication and transcription activator (RTA) gene *orf50* which, beyond the transcriptional regulation and activation of lytic DNA replication, also induces proteasome-mediated degradation of both cellular and viral proteins by its ubiquitin E3 ligase activity [53,54,55].

## 3. HHV8 Infection, Physiological Alterations and Pathogenesis

### 3.1. Endothelial Cells: Glycolysis, Warburg Effect and Oncogenesis

The induction of fatty acid synthesis and glycolysis, which represents the main source of ATP in cancer endothelial cells, is also believed to be involved in mechanisms related to energy production and cell-membrane morphogenesis, as well as cell growth stimulation and maintenance of the division rate [56,57]. More specifically, HHV8 latent infection has been reported as being characterized by the Warburg effect, which is a typical metabolic alteration in tumour cells consisting of the enhancement of aerobic glycolysis, an increase in the production of lactic acid and a consequent reduction of oxidative phosphorylation [17,58,59]. This phenomenon, which is common to most cancer cells, seems to be necessary for tumour transformation and malignant cell survival, as well as for HHV8 latency and oncogenesis in permissive cells, such as the endothelial ones which are the most relevant in KS lesions. Delgado et al. [17] suggested that the Warburg effect is crucial in creating the favourable conditions for initial tumour formation by microenvironment acidification that impairs immune system response and efficiency. Interestingly, the use of the immunosuppressive drug Rapamycin, which inhibits the mTOR pathway, is able to prevent glycolysis and can activate cell apoptosis in transplant recipients [17]. Moreover, it has been shown that most of the 200 metabolites studied undergo the same kind of alterations as cancer cells in HHV8 infected endothelial cells (see also Table 1). These include the increase in long-chain fatty acid synthesis and lipid accumulation in cytoplasmic lipid droplets (LDs), both of which are required for the biogenesis of cell membranes and for HHV8 latency in infected cells. The drugs that inhibit this pathway were able to cause cell apoptosis, providing clear proof that oncogenic viruses can modify cell metabolism [25,60] and mitochondrial phosphorylation [61]. In addition, in a recent work Wu et al. [62] stated that both cancer and diabetes could be associated with altered lactate metabolism. Another important finding was reported by Rose et al. [63] who found an increased expression of IRs in HHV8-latently infected cells. Moreover, latently infected cells are less affected by metabolic stress and drug toxicity [19], and show an increased vascular permeability [64]. Subsequently, Ingianni et al. [65] found that IR expression seemed to be normal or slightly enhanced in lytic infection, whereas an increase was already evident after 2 weeks with a remarkable over-expression of up to 130% in the late latent infection as compared to mock-infected cells. IR over-expression and the increase of insulin binding after HHV8 infection led to some important modifications in glucose metabolism which was normal or slightly depressed during lytic infection but showed an increase of up to 140% in uptake during latency. However, the biochemical basis of these metabolic alterations is not well understood. Guilluy et al. [64] and Gregory et al. [66] reported that HHV8 can trigger the PI3K/AKT/mTOR cascade in HUVEC cells and that this pathway is necessary for cell metabolism and progression. In addition, Caselli et al. [67] reported that HHV8 infection is fundamental in promoting pathogenic angiogenesis and inflammation. Angius et al. [60] performed a series of experiments to understand the role of HHV8 infection in glucose and lipid metabolism in HUVEC cells. They found an enhancement of triglyceride (TGs) synthesis during the lytic infection and normal, or slightly decreased, cholesterol esterification, whilst conversely during the latent phase of infection, TGs synthesis was sharply reduced and a strong increase in cholesterol ester (CEs) production was observed. Both TGs and CEs are synthesized and then stored in cytoplasmic LDs, which are considered to be a platform for viral morphogenesis and particle assembly for several viruses (namely HBV, GBV-B, and Dengue viruses) [60]. A remarkable increase in LDs was detected in the course of the infection from the lytic to the latent phase. Moreover, CEs seem to be required for the neo-angiogenesis that is typical of HHV8 pathogenicity in endothelial cells, representing one of the main causes responsible for the remarkable metastatic potential of KS lesions. Indeed, compounds that block the formation of CEs were found to inhibit the formation of new vessels [17,57,60,61,66]. These results support the finding that TGs synthesis and LD increase are necessary during viral replication. However, during HHV8-latency, LD content is mainly constituted by CEs, as indicated by the high rate of cholesterol esterification. High CEs and an increase in cholesterol esterification are often reported in cancer cells [68]. The storage of CEs in the LDs has been related to the increased amount of cholesterol required by malignant cells for membrane biogenesis and neo-angiogenesis [60]. Moreover, CEs have also been correlated to the severity and metastatic activity of prostate cancer [69]. As a matter of fact, angiogenesis is the major effect induced by HHV8-chronic infection and it is still present even in low-serum conditions [70]. In addition, the specific inhibition of CE synthesis was followed by a strong reduction of micro-tubules in latently infected cells [60]. Therefore, CEs seem to be strictly related to angiogenesis in the latent infection, suggesting that neutral lipids may be involved in controlling the malignant process and progression. All these findings lead to the conclusion that the enhancement of insulin binding, increased glucose uptake and the activation of other metabolic pathways may give the infected cells a metabolic advantage in terms of cell multiplication and neo-angiogenesis, and that cholesterol esterification inhibitors could be a valuable therapeutic tool for HHV8-associated malignancies [60].

### 3.2. HHV8 and Cyclooxygenase: Induction and Suppression of Immune Reaction

In a previous work, Sharma-Walia et al. [71] reported an enhanced production of Cyclooxygenase 2 (COX-2) in HHV8 infected endothelial cells and KS tissues. COX-2 was found to be important for cytokine production, cell survival and anti-apoptosis activity in HHV8 infection. Moreover, they demonstrated that the use of COX-2 inhibitors reduced HHV8 latency suggesting that anti-COX-2 drugs could be used to control HHV8 infection in KS. Although HHV8 infection in vitro can induce stable modifications in endothelial cell metabolism, the in vivo immune system tries to face HHV8 replication and diffusion by inducing a specific humoral and cellular immunological response [17,27,60,61,65]. B-lymphocytes, the main target of HHV8 infection, represent the viral reservoir and carry the virus to other body tissues. Upon infection B-lymphocytes induce several impairments of the immune system due to vIL-6 production and the induction of chronic inflammation [51,66,72]. Recently Li et al. [73] found that vIL-6, mainly localized in the endoplasmic reticulum, has a pivotal role in viral metabolism, viral lytic replication and enhancement of cell growth. It also contributes to HHV8 infectivity and pathogenesis by the stimulation of cell multiplication and induction of neo-angiogenesis. Interestingly, these authors stated that vIL-6 can regulate infected cell metabolism by upregulating mannose-6-phosphate activity. As a consequence, HHV8 was able to down-regulate immune-system function by inhibiting or silencing specific immune responses, thus favouring virus pathogenicity and spread. Caselli et al. [67] also found that HHV8 infection is important for promoting in vivo inflammation and angiogenesis in HHV8-induced lesions. Overall, the activation of COX-2 and production of vIL-6 may confer a strong physiological advantage on HHV8-infected cells favouring the malignant progression in clinical KS.

### 3.3. Immune Response and Cell Metabolism

Although HHV8 can cause frequent and severe sarcoma or other lympho-proliferating diseases in immune-depressed subjects, it is generally constrained to the latent phase in order to escape adaptive immunity. It has also been reported that HHV8 together with the activation of both humoral and cellular immunity, as well as the induction of cytokines and reactive oxygen species (ROS), induces persistent metabolic modifications in the infected endothelial cells [60,66,74,75,76,77,78]. Recently, Angius et al. [24] corroborated previous evidence [63,65] and also found that the anti-HHV8 immune response can further enhance the uptake of both glucose and insulin in latently infected endothelial cells, strengthening the hypothesis that anti-HHV8 antibodies provide additional support for virus persistence in the host [24]. The vGPCR and K1 proteins are present in a significant amount in the membranes of infected cells, whereas other proteins are preferentially localized in the cytoplasm and the nucleus. Moreover, these proteins are known to activate the PI3K pathway in latently infected BC3 lymphoblastic cells, thus enhancing HHV8 pathogenicity. It is noteworthy that PI3K has been reported to stimulate the membrane glucose transporter GLUT4 [24]. Also, mainly anti-lytic phase antibodies were significantly increased in diabetes type 2 (DMT2) patients, whilst anti-latent phase antibodies were not [24,79]. The latter authors studied the role of specific anti-HHV8 antibodies in the uptake and metabolism of insulin and glucose by HHV8-infected human endothelial cells. They used various biochemical and molecular methods to detect the expression of the pPI3K complex and the insulin and glucose uptake by HUVEC cells. They found that HHV8 induced an increase in both insulin and glucose uptake in HHV8-infected cells. Surprisingly, they also discovered that anti-HHV8 antibodies could selectively induce a further increase in insulin and glucose uptake in the latent HHV8-infection. These authors finally suggested that the immune response to HHV8-infection was able to stimulate cell metabolism by inducing an increased insulin and glucose uptake in virus infected cells, as generally happens in several tumour-viral infections [24]. As a matter of fact, when anti-HHV8 antibodies were added to endothelial cells during HHV8 latent infection, a further increase in pPI3K expression and in insulin and glucose uptake was observed. In sum, these authors hypothesized that anti-HHV8 antibodies may trigger the PI3K pathway by binding to some surface viral epitopes (e.g., K1, vGPCR), inducing a further enhancement of insulin and glucose uptake, thus enhancing HHV8 growth rate and pathogenicity.

### 3.4. HHV8 Prevalence in Chronic Diseases

The findings that HHV8 latency is characterized by persistent modifications in endothelial cell metabolism have led some authors to hypothesize a possible involvement of HHV8 infection in the development of a diffused chronic human disease such as DMT2 [65,75,78,79,80,81,82]. Although KS is considered a rare disease, HHV8 infection has been found to have a high prevalence in some countries, namely in the Mediterranean and African regions [81,82,83]. Ingianni et al. [81] were the first authors to demonstrate a strong epidemiological correlation between HHV8-infection and DMT2 patients in a Southern Italian region. Subsequently, Sobngwi et al. [82] reported a significant correlation between HHV8-infection and ketosis-prone diabetes in sub-Saharan Africa, where HHV8 is highly prevalent. Ingianni et al. [65,81] showed that about 50% of DMT2 patients were infected with HHV8, as compared to a prevalence of about 8–12% in the controls. Sobngwi et al. [82] also showed that HHV8 was able to infect pancreas β-cells, and HHV8 proteins were detectable in islet cells. They concluded that HHV8 could be associated to DMT2 since it infected pancreas β-cells, thus impairing insulin production. Piras et al. [79] have shown that HHV8 was the only Herpesvirus to be significantly correlated to DMT2, confirming this association in the Sardinian diabetic population. Furthermore, they described a HHV8 genotype with relevant genomic differences in the *orf26* and K1 genes (referred to as a “new-type”), when the isolated virus from DMT2 patients was compared to that from healthy volunteers and wild-type virus strains. Moreover, it has been reported that classic KS is more frequent in diabetics than in the normal population [84], raising the question as to whether diabetes facilitates KS or, conversely, whether HHV8 may be a risk factor for DMT2 [63,65,69,85,86]. Furthermore, Caselli et al. [75] reported a high prevalence of HHV8 specific killer-cell immunoglobulin-like receptor (KIR) allotypes in DMT2 patients. DMT2, a chronic multifactorial disorder, is a frequent complication in the elderly and represents a life-threatening social disease that affects about 6–8% of the world population. Besides the recognized risk factors such as genetics, nutrition, familiarity and environment, increasing evidence indicates that persistent microbial and viral infections may also cooperate in the development of chronic diseases like diabetes. To the best of our knowledge, to date, only HCV and HHV8 viruses have been associated to DMT2 on a biological and epidemiological basis [25,60,61,65,76,78,79,82,87,88]. In particular, HCV has been reported as causing diabetes by its direct action on liver metabolism and also by the activation of a specific immune response that may cause tissue damage, whereas a strong epidemiological association with HHV8 has been described in the last decade [65,75,78,81,82,86]. In a recent paper, Lontchi-Yimagou et al. [89] studied insulin secretion in HHV8-positive and -negative sub-Saharan African subjects with DMT2. In the diabetic population, they did not find any significant differences in metabolic parameters between patients with or without HHV8 antibodies. Yet, they found that some general metabolic parameters, namely HOMA-β, C-peptide levels and BMI, were lower in HHV8-DNA positive compared to HHV8-DNA negative DMT2 patients. Conversely, concentrations of LDL and total cholesterol increased in HHV8-DNA positive DMT2 compared to controls. TGs were also found to be increased in subjects with antibodies to HHV8 compared to those lacking HHV8-specific antibodies. The authors speculated that HHV8 can directly infect β-pancreatic cells followed by a decreased insulin secretion. In addition, the possible reactivation of the lytic virus infection can cause severe inflammation with a loss of β-pancreatic function [82]. Indeed, in HHV8, the switch from the lytic phase to latent replication is associated with a decrease in specific pancreas inflammation, with better insulin secretion and an improvement of β-pancreatic cell function. Moreover, the dysfunction of endothelial cells has been involved in the pathophysiology of metabolic diseases and DMT2 [90,91], and endothelial dysfunction is believed to contribute to the clinical expression of atherosclerosis and other complications of diabetes [92]. Overall, several findings have led to the hypothesis that HHV8 could be implicated in inducing some metabolic alterations, such as the increase of insulin binding and glucose uptake, which may be the initial event for activating the metabolic syndrome and diabetes [60,77,78,81,82,89,93]. It is commonly accepted that insulin hyper-secretion is considered a triggering cause of diabetes, and the discovery of the causes that are linked to insulin hyper-secretion is fundamental in the design of specific diabetes treatment [94,95]. The hypothesis about a possible association between HHV8 and DMT2 requires more and definite in vivo demonstrations to be confirmed. However, if this hypothesis were true, it would open a new intriguing and impactful path of research.

### 3.5. HHV8-Infection and Oxidative Stress

Latent viral infections may induce the activation of adaptive immunity with the increased production of cytokines and ROS [60,74,77,87,96]. Furthermore, the production of ROS is often accompanied by glycolysis, and reliance on ROS production is found to cause a reduction in anaerobic metabolism [90,97]. Bottero et al. [74] reported that during the early phase of endothelial cell infection, HHV8 shows an increase in viral vGPCR expression and in ROS production, with a consequent down-regulation of both innate and acquired immunity. vGPCR expression also induces ROS production in uninfected cells with an enhancement of NOX activity. Moreover, the treatment of HHV8-infected cells with the antioxidant drug N-acetylcysteine (NAC) inhibited HHV8 infection and gene transcription [74]. It should be noted that KS oncogenesis and ROS production have been associated with supporting cell proliferation and angiogenesis during the HHV8 lytic cycle [98]. In addition, inflammation and abnormal ROS production can induce endothelial dysfunction which may represent a risk factor for metabolic syndrome, hypertension and cardiovascular complications causing vascular or degenerative diseases [66,75]. In actual fact, recent studies have suggested a physiological role of cellular metabolism in vessel sprouting, and endothelial cells are generally dysregulated in pathological conditions and cancer [99]. Very recently, Incani et al. [77] corroborated the assumption that DMT2 is associated to plasma oxidative stress [100], and a similar condition has also been reported in HHV8-infected subjects wherein the HHV8-infection, by inducing abnormal ROS production, most probably contributes to causing and/or maintaining a status of oxidative stress [77] and hence tissue damage. As a consequence, plasma lipid oxidation contributes to endothelial cell dysfunction, which characterizes the onset of atherosclerotic plaque and blood vessel disorders [64,74,101,102,103].

## 4. Concluding Remarks and Future Scenarios

**Figure 1:**
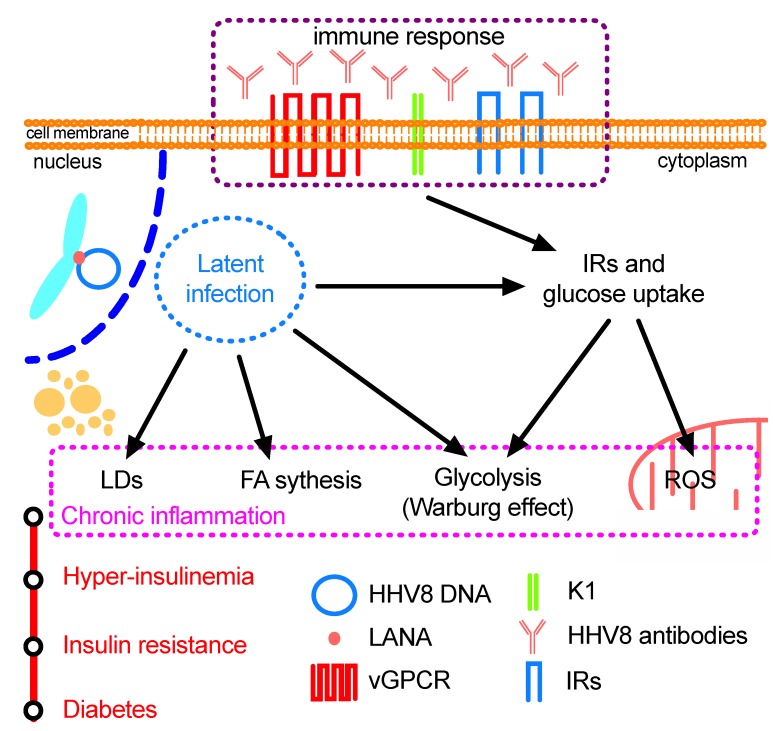
Schematic representation of the HHV8-induced metabolism alteration of endothelial cells and the hypothetical association with diabetes type 2 development. Latent-persistent HHV8 infection, together with the humoral immune response, induces a strong enhancement of insulin receptors (IRs) with a consequent increase in glucose uptake that skews the cell metabolism toward the anaerobic glycolysis that leads to lactate accumulation (Warburg effect). The higher glucose concentration and the hypoxic condition trigger mitochondrial ROS production. Moreover, the latent infection also stimulates fatty acid (FA) synthesis and the accumulation of neutral lipids in cytoplasmic lipid droplets (LDs). Overall, in a body metabolism context, these alterations may evolve into chronic systemic inflammation with the higher IRs expression further stimulating insulin production by pancreatic beta-cells and the feasible induction of hyper-insulinemia that, time by time, may lead to insulin resistance and hence to diabetes type 2.

As already stated, one of the most peculiar features of HHV8 infection is its tropism for endothelial cells and B-lymphocytes. While chronic infection of B-cells can lead to an impairment of the immune response [66], despite favouring the persistence and spread of the virus, the infection of endothelial cells may have a profound effect on the host’s metabolism, since it is strictly involved in the first step of food processing and in lipid metabolism [90]. Several studies indicate that endothelial dysfunction can be implicated in metabolic disorders and even in DMT2 [90,94,95]. In particular, in HHV8-infected endothelial cells, ATP production is mainly accomplished by glycolysis as often happens in tumour cells; interestingly, anaerobic metabolism is also accompanied by enhanced ROS production which has been found to be involved in atherosclerosis, insulin resistance and diabetes. In addition, it has already been reported that ROS may also have a direct fundamental role in the metabolic modification leading to DMT2, and agents that increase or generate ROS can stimulate basal insulin secretion [94]. It is worth noting that insulin resistance has been reported to be at the basis of DMT2, and that all the factors that induce hyper-insulinemia may cause insulin resistance [94,104]. Recent research on HHV8 infection has revealed that IRs are over-expressed, enhancing glucose uptake and utilization, and TGs synthesis is increased during the lytic phase of infection facilitating virion morphogenesis and vessel neo-angiogenesis; during the latency phase, the virus switches to an increased synthesis of the CEs required for tumour cell transformation and multiplication. Therefore, it seems that the virus has evolved in order to express a very limited number of genes essential for latency, angiogenesis and cell transformation. However, we are still far from elucidating exactly how the host reacts. Primarily, it tries to produce humoral immunity, but the antibodies are not able to neutralize the intracellular virus, and de facto they seem to further support viral persistence by over-stimulation of IR expression and enhancing glucose uptake. At the same time, cellular immunity is affected by the chronic infection of the B-lymphocytes which are unable to get rid of the hidden virus. All these conditions allow the virus to efficiently persist throughout the host’s lifespan, with the possibility of occasionally switching to the lytic phase, in case new viral progenies are required to spread the infection further. As a consequence of the chronic infection, the host presents a general metabolic disturbance, with an alteration of both glucose and lipid metabolism, impairment of the general immune system and enhancement of ROS production, which can evolve into severe KS disease when other disorders can further decrease the efficiency of the immune system. However, researchers have started to focus their interest on some new and very important findings in chronic HHV8 infection: i) HHV8 can infect pancreas beta-cells, with a possible decrease in insulin production; ii) the virus infection leads the host to use abnormal amounts of insulin and glucose; and iii) the host produces specific antibodies which further stimulate insulin and glucose utilization. On the basis of these considerations, some authors have hypothesized a possible association between chronic HHV8 infection and the onset of chronic diseases such as diabetes. The recent studies performed in the Mediterranean and sub-Saharan Regions have given significant evidence of an epidemiological association between HHV8 infection and DMT2 [77,78,86,89]. Furthermore, the upset of both the glucose and lipid metabolism of endothelial cells has been hypothesized as creating the conditions that favour the metabolic syndrome and decrease in insulin efficiency found in diabetes (Figure 1). Although these hypotheses are intriguing and stimulating, more research is needed to confirm a possible association between HHV8 infection and DMT2. Some crucial questions must be properly answered: a) do the glucose and lipid metabolism change and how are they changed in those cells that consume most of the insulin and glucose in the human organism, such as myocytes and adipocytes? b) In view of the fact that some of the proteins produced during viral latency share analogous epitopes with host proteins, what effects could antibodies against these proteins have on cell metabolism? The answers to these questions will open a very fruitful field of speculation and research.

## Figures and Tables

**Table 1:** Modification of some biochemical parameters in HHV8 infected endothelial cells.

Metabolites	HHV8 Lytic Infection	HHV8 Latent Infection
* Cholesterol esters	−	+++
** Fatty acids	++	−
** Spermidine	−	++
** 7-beta-hydroxycholesterol	−	+
** Mannose-6-phosphate	−	++
* Glucose uptake	−	+++
** phospho-enol-pyruvate	−	+++
** 6-phosphogluconate	−	+
* Triglycerides	+++	−

* from Angius et al. [60]; ** Delgado et al. [25]. The sign − means a normal or a slight decrease of the metabolic parameter as compared to control; the signs +, ++ and +++ indicate a progressive increase of the metabolites in infected cells versus uninfected controls.
